# Immunoglobulin A/PIGR axis as potential mediators of human abdominal aortic aneurysms revealed by topologically resolved proteomics

**DOI:** 10.1186/s12967-025-06758-y

**Published:** 2025-07-07

**Authors:** Isabel Cerro-Pardo, Estefanía Núñez, Belén Picatoste, Cristina Márquez-Gálvez, Lucía Ortega-Villanueva, Irene Raposo-Gutiérrez, Jes S. Lindholt, Luis Miguel Blanco-Colio, Almudena R. Ramiro, Jesús Vázquez, José Luis Martín-Ventura

**Affiliations:** 1 Vascular Lab, Instituto de Investigación Sanitaria Fundación Jiménez-Díaz- Autónoma University of Madrid (IIS-FJD, UAM), Av. Reyes Católicos 2, 28040 Madrid, Spain; 2 CIBER de Enfermedades Cardiovasculares (CIBERCV), Madrid, Spain; 3 Cardiovascular Proteomics Lab, Centro Nacional de Investigaciones Cardiovasculares (CNIC), Madrid, Spain; 4 Hepatic and Vascular Diseases Laboratory, Biochemistry and Molecular Biology Department, School of Pharmacy, Complutense University of Madrid, Madrid, Spain; 5 B Cell Lab, Centro Nacional de Investigaciones Cardiovasculares (CNIC), Madrid, Spain; 6 Centre for Individualized Medicine in Arterial Diseases, Odense University Hospital, Odense, Denmark

**Keywords:** Abdominal aortic aneurysm, Immune response, Proteomics, Biomarkers, Immunoglobulin A, Polymeric immunoglobulin receptor

## Abstract

**Background:**

Abdominal aortic aneurysm (AAA) is an asymptomatic chronic disease of the aorta and its evolution is unpredictable. Despite the existence of several pathological mechanisms contributing to the dilation of the human AAA wall, there is currently no specific therapy to prevent the fatal rupture of the aorta. Our objective was to identify novel mediators and/or biomarkers involved in the instability of the aortic wall that could help to prevent AAA progression.

**Methods:**

Multiplexed quantitative proteomic analysis of human AAA and healthy aortic wall (medial and adventitial layers) was performed. Results were subsequently validated by western blot and immunohistochemistry, as well as by turbidimetry/ELISA of tissue-conditioned media. In addition, immunoglobulins A1 and A2 (IGA1 and IGA2) plasma levels were analyzed by turbidimetry in a pilot study [controls (n = 22) and AAA patients (n = 22)] and in a validation study with a 6-year follow-up [controls (n = 64) and AAA patients (n = 189)]. In vitro experiments were performed in THP-1-derived macrophages (basal or polarized to M1 or M2). Polymeric immunoglobulin receptor (*PIGR*) mRNA expression and secretion in macrophages were analyzed by Q-PCR and ELISA, respectively. Finally, the hematopoietic contribution of PIGR was assessed in experimental AAA (*Ldlr*^*−/−*^ mice fed an atherogenic diet and 1 μg/Kg/min angiotensin II infusion for 28 days) by bone marrow transplantation experiments.

**Results:**

Functional analysis of biological pathways altered in human AAA wall revealed a significant upregulation of components of the adaptive immune response, including IGHA1 and IGHA2, as well as the IGA receptor, PIGR. In addition, IGA2, but not IGA1, plasma levels were significantly increased in a pilot study of AAA patients relative to controls (489 ± 38 vs 344 ± 36 mg/L, p < 0.01). This finding was further validated in a larger cohort, confirming the association of IGA2 with AAA presence independent of risk factors and treatments [OR = 2.140 (1.109–4.130), P < 0.05]. Furthermore, in the validation cohort, elevated IGA2 plasma levels were independently associated with AAA progression [HR = 1.941 (1.108–3.399), p < 0.05]. PIGR colocalized with macrophages in the AAA wall and, *PIGR* mRNA levels were increased following the differentiation of THP-1 monocytes into macrophages, as well as in M1-polarized THP-1 macrophages compared to M2 macrophages. *Pigr* deficiency in hematopoietic cells resulted in a significantly reduced AAA incidence (14 vs 57%) and decreased macrophage infiltration (3.5 ± 0.5 vs 5.6 ± 0.7%).

**Conclusions:**

Increased IGA and PIGR is observed in the AAA wall. *Pigr* deficiency in hematopoietic cells decreases AAA progression, suggesting a therapeutic role for PIGR in AAA.

**Supplementary Information:**

The online version contains supplementary material available at 10.1186/s12967-025-06758-y.

## Background

Abdominal aortic aneurysm (AAA) is a life-threatening disease that results from the chronic dilation of the abdominal aorta when it exceeds 50% of its diameter. The prevalence of AAA is highest among men aged 65 to 75 years who have ever smoked, occurring in approximately 7% of this population [1]. Other risk factors for AAA include having high blood pressure or a family history of AAA [2]. The majority of AAAs are asymptomatic until rupture occurs. Consequently, only those incidentally identified (or screened) by imaging techniques are diagnosed in the early stages (3–5 cm). In such cases, follow-up by ultrasound is the strategy recommended [3]. Currently, surgical repair of AAAs is only advised when the aortic size is higher than 5 or 5.5 cm, either through open surgery or endovascular repair [3]. However, AAA evolution is unpredictable given that it comprises periods of growth and stability [4]. This, coupled with the dearth of efficacious therapeutic strategies for AAA, underscores the imperative for a more comprehensive understanding of AAA pathology to identify novel diagnostic, prognostic and/or therapeutic targets.

The vascular remodelling process involved in the pathogenesis of AAA is characterized by the interaction of resident cells (endothelial and vascular smooth muscle cells -VSMC-) and circulating cells (monocytes, platelets, neutrophils and erythrocytes), along with systemic/blood components retained in AAA [3, 4]. Specifically, interactions between cells with proteases and cytokines result in a proteolytic and oxidative injury to the aortic wall, which favours the phenotypic switch and eventual disappearance of VSMC in the medial layer, along with an immune-inflammatory response in the adventitial layer. Despite the extensive description of these processes in human AAA, their counterparts in experimental models remains limited, hindering the translation of findings into clinical applications. As an alternative to research using experimental models, some studies have applied non-hypothesis-driven high-throughput proteomic approaches to human AAA samples, including plasma, circulating cells or tissue-secretome with the aim to search for novel biomarkers of AAA [5–7]. Similarly, proteomic studies of AAA wall homogenates have also been employed to identify novel mechanisms involved in AAA pathogenesis [8–10]. However, while these studies on AAA tissues yielded valuable insights, they all analyzed full-thickness samples, thereby failing to distinguish between medial and adventitial processes. In this respect, the media layer of AAA involves processes mainly related to VSMC metabolism, while the adventitial layer of AAA is much more complex due to the involvement of different types of cells, such as T- and B-cell aggregates, macrophages, adipocytes, fibroblasts, along with nerves and neovessels. Thus, the novelty of this paper lies in the approach, which aims to analyze the medial and adventitial layers separately, attempting to distinguish between processes related to AAA dilation (mainly involving the media) and/or instability (mainly involving the adventitia). Consequently, in this work, mass spectrometry-based proteomics was used to compare aorta homogenates from the medial and adventitial layers of human AAA and healthy individuals to identify novel mediators involved in the progression and/or rupture of AAA.

## Materials and methods

The study setup aim, design and setting is shown in a schematic diagram in Supp. Figure 1.

### Human plasmas

This observational study was conducted as part of a population-based image screening trial for AAA in Danish men aged 65–74 years between October 2008 and October 2010 (VIVA, ClinicalTrials.gov NCT00662480). The design of the protocol has been described in detail previously [11]. In summary, the VIVA AAA cohort comprised cases of AAA diagnosed through population-based screening in the VIVA trial, which randomized more than 50,000 men aged 65–74 1:1 to either vascular screening for AAA, peripheral arterial disease and hypertension, or a control group. Cases with AAA were recommended a daily dose of 40 mg simvastatin and low-dose aspirin and offered AAA repair if their AAA was 55 mm or more in diameter, and annual follow-up if smaller. Blood samples were collected from the patient during a study consultation for information and the initiation of cardiovascular disease (CVD) prevention measures.

A preliminary study was conducted with 22 cases and 22 controls, followed by a validation study involving 64 controls and 189 cases with available blood samples and a 6-year follow-up. No additional exclusion criteria were employed, except in cases where blood sampling could not be performed for logistic reasons, unsuccessful blood sampling, or when samples were exhausted. The study was conducted and reported in accordance with the STROBE recommendations. All subjects provided informed consent, and the study was approved by local ethics committee of the Viborg Hospital (M20080025) and performed in accordance with the Helsinki Declaration.

### Human tissues

Abdominal aortas were collected from brain-deceased organ donors during organ removal for therapeutic transplantation (kidney or liver transplantation) with the authorization of the French Biomedicine Agency (PFS 09-007, BBMRI network, BB-0033-00029). Prior to participation, ethical committee advice and patient written informed consent were obtained (RESAA and AMETHYST studies, CPP Paris-Cochin 2095, 1930, and 1931, INSERM Institutional Review Board, IRB0000388). All human studies were conducted in accordance with the principles outlined in the Declaration of Helsinki. The aortic tissue was washed and preserved in Ringer's lactate solution at 4 °C until required for use. Following a macroscopic examination, the aortas devoid of atheromatous lesions were classified as control aortas. In addition, AAA walls were obtained from patients who were undergoing surgical repair of AAA enrolled in the Reflet Sanguin de l'evolutivite des Anevrysmes de l'Aorte abdominale protocol. A small portion of tissue from each sample was fixed in 3.7% paraformaldehyde for classical histology and immunohistochemistry assessments. Both the AAA and control aortas were incubated in culture medium to obtain tissue-conditioned media or placed in liquid N_2_ for subsequent protein extraction (homogenates).

For tissue-conditioned media, samples were cut into small pieces (5 mm^2^) and incubated in RPMI 1640 medium devoid of proteins, containing antibiotics/antimycotic (Gibco) for 24 h at 37 °C (6 mL/g of wet tissue). The conditioned media (supernatants containing proteins released by the tissue samples) were obtained following centrifugation (3000 g, 10 min, 20 °C) and stored at − 80 °C until further processing.

### Human cells

The human monocytic THP-1 cell line was purchased from ATCC (TIB-202) and cultured in RPMI-1640 medium (Sigma) supplemented with 10% decomplemented (heat-deactivated) fetal bovine serum (FBS; Sigma), 2 mM L-glutamine and 100 U/mL penicillin and 100 mg/mL streptomycin (all purchased from Sigma) at 37 °C in a humidified 5% CO_2_ atmosphere. For experiments, THP-1 monocytes were differentiated into macrophages by incubating them for 24, 48 or 72 h with 100 nM phorbol 12-myristate 13-acetate (PMA, Sigma, P8139) in medium containing 10% FBS. Following a 72-h differentiation period, macrophages were stimulated with TNF-α (100 ng/mL) for 24 h to test *PIGR* mRNA expression. In addition, following a 72-h differentiation period, macrophages were polarized into M1 macrophages by incubation with 20 ng/ml of interferon gamma (IFN-γ, 300-02, Peprotech) and 100 ng/ml of Lipopolysaccharide (LPS, 8630, Sigma) or into M2 macrophages by incubation with 20 ng/ml of interleukin-4 (IL-4, 200-04, Peprotech) and 20 ng/ml of IL-13 (200-13, Peprotech) in medium containing 0.5% FBS for 24 (for mRNA analysis) or 48 h (for secretion analysis).

### Experimental model

For bone marrow (BM) transplantation experiments, 10-week-old male CD45.1 *Ldlr*^*−/−*^ mice (n = 28) were subjected to medullar aplasia with two doses of 5.5 Gy total body irradiation separated by 4 h. The following day, femurs and tibias from 6-week-old female CD45.2 *Pigr*^*−/−*^ (30988, MMRRC) or *Pigr*^+*/*+^ mice were harvested and processed to obtain bone marrow cell suspension. 2.5 × 10^6^ bone marrow pooled cells from each genotype donors were injected intravenously into the tail veins of irradiated mice (n = 14 for each group) to rescue their hematopoietic system. Mice were maintained on antibiotic water for 4 weeks after irradiation, then placed on regular water. 4 weeks after transplantation, bone marrow reconstitution was checked in blood using the CD45.1 and CD45.2 haplotypes as markers to monitor the engraftment of donor cells within the chimeric recipient. CD45 allelic variants were detected on blood leukocytes with fluorescence-activated-cell sorter (FACS) using fluorescently labeled monoclonal antibodies (17–0453-82 for CD45.1 and 45–0454-82 for CD45.2). Samples were acquired on LSR Fortessa instruments (BD Biosciences) and analyzed with FlowJo V10.4.2 software. Then, mice were fed a Western-type diet for 5 weeks. Mini-osmotic pumps (ALZET® 2004) containing Angiotensin II (1000 ng/kg/min) were implanted subcutaneously for 28 days 1 week after the initiation of the diet. Mouse aortic samples were embedded in paraffin. Serial sections (4 μm) of aortas were cut for histomorphometry. Histomorphometry was performed on hematoxylin–eosin stained histological sections. For AAA incidence, changes in suprarenal aortic diameter were calculated as percentage of dilation over baseline at day 28 and AAA incidence was quantified based on a definition of an increase in aortic diameter of more than 50% over the baseline according to previously reported methods [12]. Images were taking using a Leica DMD108 Microscope.

### Proteomics

#### Protein digestion, TMT labeling and peptide fractionation

Human AAA tissues were subjected to tissue homogenization with ceramic beads (MagNa Lyser Green Beads, Roche, Germany) in CS buffer (Pipes pH6.8, MgCl2, NaCl, EDTA, sucrose, SDS, sodium orthovanadate; Biochain Institute, Inc. #K3013010-5) freshly supplemented with protease and phosphatase inhibitors and in the presence of 50 mM Iodoacetamide (IAM). Extracted proteins from tissue samples (~ 200 μg) were subjected to in-filter reduction and alkylation using 50 mM S-methyl methanethiosulfonate (MMTS) followed by trypsin digestion (Nanosep Centrifugal Devices with Omega Membrane-10 K, PALL), and the resulting peptides were TMT-labeled (Thermo Fisher Scientific) following manufacturer’s instructions. For the medial layer, we compared nine different samples from healthy walls with nine from AAA walls; the same number of biological replicates were used for the adventitial layer. One channel in each TMT experiment was reserved for reference internal standard, which was created by pooling healthy samples. Labeled peptides were fractionated using the high pH reversed-phase peptide fractionation kit (Thermo Fisher Scientific) according to manufacturer’s instructions. Briefly, cartridges were washed with 50% and 100% acetonitrile (ACN) and equilibrated with 0.1% of TFA (Trifluoroacetic acid). 100 µg of peptides were resuspended in TFA 0.1% and loaded into the cartridges. Peptides were then eluted into five fractions with increasing amounts of ACN: Fr1 (12.5% ACN), Fr2 (15% ACN), Fr3 (17.5% ACN), Fr4 (20% ACN) and Fr5 (50% ACN).

### LC–MS/MS analysis

Each fraction of labeled peptide samples was analyzed using an UPLC-Ultimate 3000 (Thermo Fisher Scientific) coupled to a Q-exactive HF Hybrid Quadrupole-Orbitrap (Thermo Fisher Scientific) using a PepMap 100 C18 0.3 × 5 mm ID as trapping column (Thermo Fisher Scientific) and a PepMap RSLC C18 EASY-Spray column 50 cm × 75 mm ID as analytical column (Thermo Fisher Scientific). Peptides were loaded in buffer A (0.1% of formic acid in water (v/v)) and eluted with a 240 min linear gradient of buffer B (100% ACN, 0.1% formic acid (v/v)) at 200 nl/min. Mass spectra were acquired in data-dependent manner, with an automatic switch between MS and MS/MS with a “Top-15” method and 40 s dynamic exclusion. MS spectra were acquired in the Orbitrap analyser using full ion-scan mode with a 390–1700 m/z range and 60,000 FT resolution. The automatic gain control target was set at 1 × 106 with 50 ms maximum injection time. HCD fragmentation was performed at 30% of normalized collision energy and MS/MS spectra were analyzed at a 30,000 resolution in the Orbitrap with automatic gain control target set at 1 × 105 and 100 ms maximum injection time.

### Protein identification and quantification

For peptide identification, MS/MS spectra were searched using the SEQUEST HT algorithm implemented in Proteome Discoverer 2.5 (Thermo Scientific) against a Swiss-Prot database comprising human protein sequences (September 2015). Trypsin digestion was set with a maximum of 2 missed cleavages. TMT labeling at the N-terminal end and Lys (229.162932 Da) were set as fixed modifications, and Met oxidation (15.994915 Da), Cys carbamidomethylation (57.021464 Da) or methylthiolation (45.987721 Da) as dynamic modifications. Precursor mass tolerance was set at 800 ppm, fragment mass tolerance at 0.03 Da and precursor charge range to 2–4. The false discovery rate (FDR) was calculated using the corrected Xcorr score (cXcorr) [13] and the target/decoy competition strategy applying the picked FDR method at the peptide level [14], with an additional filter for precursor mass tolerance of 15 ppm [15]. A 1% FDR was employed as the criterion for peptide identification. Quantitative information from TMT reporter intensities was integrated from the spectrum level to the peptide level, and then to the protein level based on the weighted spectrum, peptide and protein (WSPP) model [16, 17] using SanXoT software package [18, 19]. Quantitative protein values are expressed using the standardized variable Zq (i.e., normalized log2-ratios expressed in units of standard deviation according to the estimated variances). Functional enrichment was performed using DAVID Gene Functional Classification Tool and STRING v12.0, and clusters were drawn with Cytoscape v3.9.1.

### Protein extraction and immunoblotting

Human AAA tissues, as well as aortic wall tissues from healthy human donors, were collected and incubated with lysis buffer containing 10 mM Tris–HCl pH 7.4 buffer, 150 mM NaCl, 0.5% NP-40, 1% Triton X-100, 1 mM EDTA, 1 mM EGTA, 10 mM NaF, 1 mM DTT, 1 mM PMSF and protease and phosphatase inhibitors and pelleted. After determining protein concentration using the Pierce BCA Protein Assay Kit (Thermo Fisher Scientific), equal amounts of protein (20 µg) were subjected to sodium dodecyl sulphate (SDS) polyacrylamide gel electrophoresis and subsequently transferred to a nitrocellulose membrane (Bio-Rad). After blocking with 5% BSA in TBS containing 0.1% Tween-20, blots were incubated overnight with one of the following antibodies: anti-human IGA1 and IGA2 (9130-01 and 9140-01, Southern biotech), PIGR (AF2717, R&D) or anti-human GAPDH (MAB374, Sigma) at 4 °C. Blots were then washed and incubated with the appropriate horseradish peroxidase (HRP)-conjugated secondary antibody for one hour and visualized using the ECL substrate kit (Thermo Fisher Scientific). The membranes were then subjected to densitometry (Image J), and values were normalized against GAPDH.

### Immunoturbidimetry and enzyme-linked immunosorbent assay

IGA1 and IGA2 levels were quantified in tissue-conditioned media from AAA walls and control aortas and in plasma using commercially available assays (The Binding Site) on the OPTILITE turbidimeter (The Binding Site) following the manufacturer’s recommendations.

PIGR levels were quantified in tissue-conditioned media from AAA walls and control aortas, as well as in the cell-conditioned media, using a commercially available ELISA kit (ab282302, Abcam) according to the manufacturer's protocol. In summary, the plates were coated with the samples or standards, followed by the antibody cocktail (capture and detector antibodies). After incubation at room temperature for 1 h, the wells were washed to remove unbound material and TMB Development Solution was added. The optical density at 450 nm was measured. After factoring sample dilutions, the original PIGR concentrations were finally calculated.

### Immunohistochemistry and immunofluorescence

For immunohistochemical analysis of IGA1, IGA2 and PIGR and for immunofluorescence analysis of PIGR in aortic paraffin sections, antigen retrieval (0.01 M citrate pH 6, 96 °C, 20 min), blockade of endogenous peroxidase (3% H_2_O_2_ in methanol, 30 min) and nonspecific binding (6% host serum, 1 h) was performed prior to incubation with the primary antibodies overnight at 4 °C (anti-human IGA1 and IGA2, 9130–01 and 9140–01, respectively, Southern Biotech, and anti-human PIGR, ab96196, Abcam)Then, samples were rinsed in PBS and incubated 1 h with goat anti-mouse and donkey anti-rabbit (HRP-conjugated, Jackson Immunoresearch) secondary antibodies. Slides were then incubated with 3,3'-diaminobenzidine (DAB) peroxidase substrate, counterstained with hematoxylin, dehydrated, mounted with DPX and examined under a light microscope (Zeiss). For immunofluorescence analysis of CD68 and alpha-smooth muscle active (α-SMA) in mouse aortic paraffin sections, frozen sections were fixed in 10% formalin for 7 min and then washed under running water for 7 min prior to primary antibody incubation (anti-mouse CD68, ab5344, Abcam, and anti-mouse α-SMA Cγ3 conjugated, C6198, Sigma). After the incubation for 1 h away from light with species-specific secondary fluorescent conjugated antibodies, nuclei were stained with 4′,6-diamidino-2-phenylindole (DAPI) and slides mounted with fluorsave medium.

Computer-assisted morphometric analysis was performed with Image J software and the Image-Pro Plus software. The threshold setting for area measurement was equal for all images. In human samples, three different positive fields were analyzed in each subject and results were expressed as % positive staining/area (IGA1, IGA2, PIGR). In animal samples, slides from each mouse were examined in a blinded manner and results were expressed as % positive area versus total area (CD68 and SMA).

For immunofluorescence, sections were fixed in 10% formalin for 7 min and then washed under running water for 7 min prior to blocking endogenous peroxidase and primary antibody incubation (anti-human PIGR, ab96196, Abcam and anti-human CD68, M0814, Dako). After the incubation for 1 h away from light with species-specific secondary fluorescent conjugated antibodies, nuclei were stained with 4′,6-diamidino-2-phenylindole (DAPI) and the slides were mounted with fluorsave medium. Stained tissues were examined using fluorescence microscopy and images were obtained using a Zeiss microscope.

### RNA isolation and RT-qPCR analysis

Total RNA was extracted from THPs using the TRIzol method (Life Technologies), and its concentration was determined by measuring the absorbance at 260 nm. A total of 1 µg of RNA was used for reverse transcription with the High-Capacity cDNA Archive Kit (Applied Biosystems). Real-time polymerase chain reaction (PCR) was performed on an ABI Prism 7500 sequence detection PCR system (Applied Biosystems), according to the manufacturer's protocol. The expression of the target genes was normalized to that of the housekeeping transcript [glyceraldehyde-3-phosphate dehydrogenase (GAPDH)]. All measurements were performed in duplicate. The amount of target mRNA in samples was estimated by the 2 ΔCT relative quantification method. The values of each sample were normalized with respect to the basal condition, expressed in arbitrary units. The following PCR primers and TaqMan probes were purchased from Applied Biosystems and optimized according to the manufacturer's protocol: human *PIGR* (Hs00922561_m1, Thermo Fisher Scientific), human CD68 (Hs02836816_g1, Thermo Fisher Scientific), human TNF-α (Hs00174128_m1) and human *GAPDH* (4310884E, Thermo Fisher Scientific).

### Statistical analysis

Results are expressed as mean ± the standard error of the mean (SEM). In vitro experiments were replicated at least three times and analyzed by Student's t test. For proteomics analysis, adjustment for multiple hypothesis testing was performed by controlling for the False Discovery Rate (FDR). Data from the experimental model were analyzed by comparing groups using the Mann–Whitney test (for AAA size and % of CD68 and α-SMA) or chi-square test (for AAA incidence). For analysis of data from human samples, groups were compared using the Mann Whitney test. The association between IGA2 and maximal anterior–posterior aortic diameter was studied by Spearman correlation analysis. To evaluate the independent association of elevated IGA2 (tertiles 2 and 3) with AAA presence, a multivariate logistic regression analysis was performed adjusted by confounding factors (smoke, body mass index-BMI-, previous CVD and aspirin). Moreover, to test whether elevated IGA2 levels were associated with a higher risk of developing aortic diameter enlargement requiring surgical intervention, a Cox regression analysis was performed. Statistical analysis was performed using GraphPad Prism (version 8.0.2 for Windows, GraphPad Software). Significance was accepted at the level of P < 0.05 (two-tailed).

## Results

### Proteomic analysis of human AAA wall

**Fig. 1:**
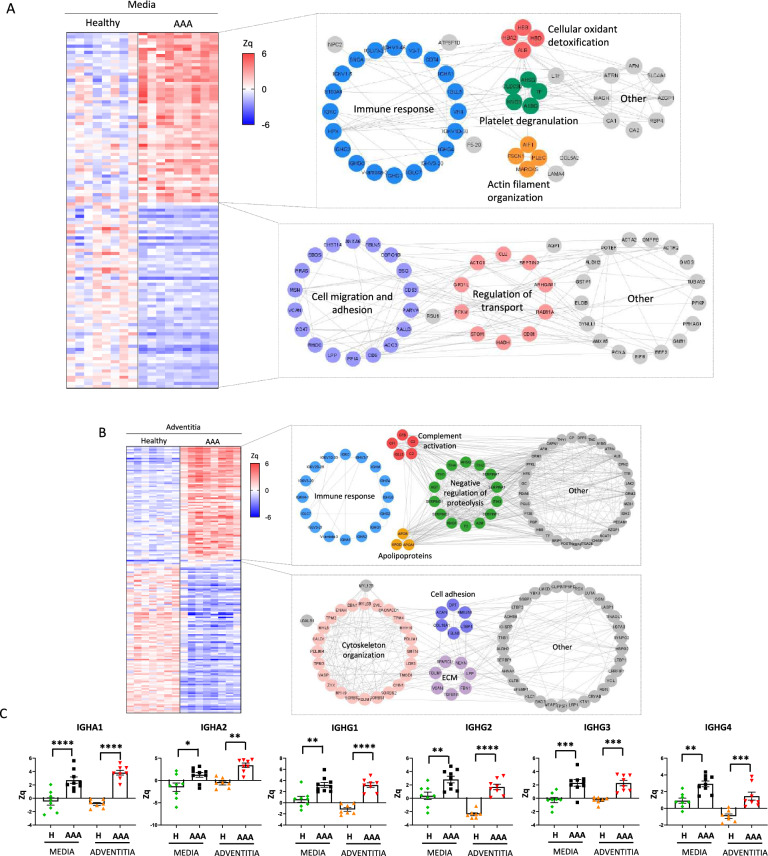
Differentially regulated proteins in human AAA wall. Heat map depicting significant protein abundance changes (FDR < 5%) in the AAA media **(A)** and adventitial **(B)** layers compared to healthy control aortas. Increased (red) or decreased (blue) abundances are shown according to the indicated Zq scale. Correlation network obtained after functional analysis of the proteins differentially expressed in the medial and adventitial layer of human AAA aortas are displayed in the right panels. **(C)** Box plots of immunoglobulins increased in human AAA adventitial layer in comparison to healthy control aortas. Distribution of protein abundances (Zq values) are plotted. *p < 0.05, ** p < 0.005, *** p < 0.0005, **** p < 0.0001. I*GHA1* immunoglobulin heavy constant A1, *IGHA2* immunoglobulin heavy constant A2, *IGHG1* immunoglobulin heavy constant G1, *IGHG2* immunoglobulin heavy constant G2, *IGHG3* immunoglobulin heavy constant G3, *IGHG4* immunoglobulin heavy constant G4

A hypothesis-free proteomics approach was employed to compare the protein profiles of medial and adventitial layers from human AAA with those from healthy individuals. Among the 3,005 and 3,263 proteins quantified, respectively, in AAA medial and adventitial layers, 115 proteins in the media (55 up and 60 down) and 363 in the adventitia (158 up and 205 down) significantly changed (FDR < 5%) their relative abundance when compared to healthy controls (Fig. 1A, B). Subsequently, a functional analysis with STRING was conducted to elucidate the roles of these proteins in both regions. The biological processes that exhibited the higher increase in the medial layer of AAA were associated with the immune response, in addition to cellular detoxification, platelet degranulation and actin filament organization (Fig. 1A). The proteins that were downregulated in the media were significantly enriched in biological categories mostly related to cell migration and adhesion and regulation of transport (Fig. 1A)**.** Proteins that were upregulated in the adventitial layer of AAA were primarily associated with the immune response and complement activation, as well as negative regulation of proteolysis and lipid transport (apolipoproteins) (Fig. 1B). In contrast, the downregulated processes were related to cytoskeleton organization, cell adhesion and extracellular matrix components (Fig. 1B). Furthermore, functional enrichment analysis of proteins significantly increased (FDR < 0.05) in the medial and adventitial layers of AAA, indicating that these proteins were predominantly involved in adaptive immune responses (Supp. Figure 2**)**. Among them, the most upregulated proteins in both, medial and adventitial layers of AAA, were immunoglobulins from IGA (IGHA1, IGHA2) and IGG (IGHG1-4) subclasses (Fig. 1C).

### IGA1 and IGA2 levels are increased in human AAA wall

**Fig. 2:**
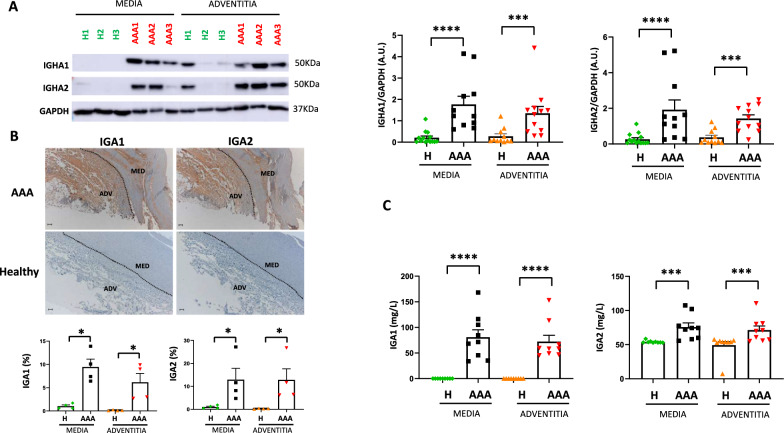
IGA1 and IGA2 levels are increased in human AAA wall. **A** Western blot and densitometric analysis of IGHA1 and IGHA2 after correction for GAPDH (loading control) in medial and adventitial layers of human AAA and healthy aortas (healthy media n = 14; AAA media n = 11; healthy adventitia n = 11; AAA adventitia n = 12). **B** Representative immunohistochemistry and quantification of IGA1 and IGA2 in serial aortic cross-sections from AAA and healthy aortas (n = 4 for both). **C** Quantification by immunoturbidimetry of IGA1 and IGA2 concentrations (mg/L) in the tissue-conditioned media of AAA and healthy aortas (healthy media n = 9; AAA media n = 9; healthy adventitia n = 9; AAA adventitia n = 9). Data represent means ± SEM. Mann–Whitney U test for healthy media vs. AAA media and healthy adventitia vs. AAA adventitia in (**A**) and (**B**). ***p < 0.001, ****p < 0.0001. Scale bars, 100 µm. A.U., arbitrary units. *IGHA1* immunoglobulin heavy constant A1, *IGHA2* immunoglobulin heavy constant A2, *IGA1* Immunoglobulin A1 (including both heavy and light chains), *IGA2* Immunoglobulin A2 (including both heavy and light chains)

IGHA1 and IGHA2 protein expression in the medial and adventitial layers of human AAA wall and healthy control aortas was validated by western blot. In accordance with the proteomics analysis, a statistically significant increase of both IGHA1 and IGHA2 expression was observed in the two layers from AAA wall compared to healthy aortas (Fig. 2A). Furthermore, the evaluation of IGA1 and IGA2 expression and localization by immunohistochemistry in human AAA and control aortas revealed a strong staining in the pathological arteries, while only a faint signal was detected in the healthy aortas (Fig. 2B). Additionally, the analysis of IGA1 and IGA2 secretion by immunoturbidimetry in AAA and control tissue-conditioned media from both the media and adventitia further confirmed increased IGA1 and IGA2 levels (Fig. 2C, p < 0.05 in both layers).

### IGA2 plasma levels predict AAA evolution

**Fig. 3:**
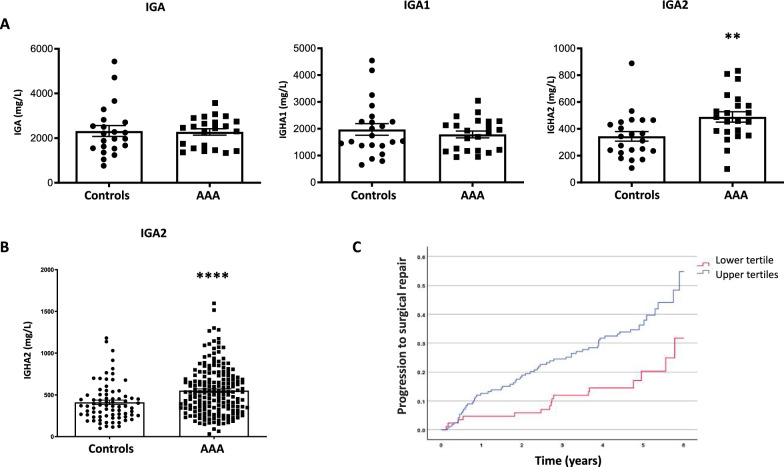
IGA2 plasma levels predict AAA evolution. **A** Total IGA, IGA1 and IGA2 concentration (mg/L) in plasma from AAA patients (n = 22) and controls (n = 22). **B** IGA2 concentration (mg/L) in plasma from AAA patients (n = 189) and controls (n = 64). **(C)** Cumulative Kaplan–Meier estimates with 95% confidence intervals for progression to surgical repair stratified by the lower and upper tertiles of IGA2 (mg/L) in AAA patients. Data represent means ± SEM. Mann–Whitney U test. ****p < 0.0001

Given the accumulation of IGA1 and IGA2 in the human AAA wall, we wondered whether this accumulation was also reflected in the circulating levels of these proteins in plasma. Thus, we measured IGA1 and IGA2 plasma levels by immunoturbidimetry from a pilot cohort including 22 AAA patients (aortic diameter > 3 cm) and 22 controls (aortic diameter < 3 cm) with no differences in age and sex. The results showed that there was a significant increase of IGA2 levels in AAA patients relative to controls (489 ± 38 vs 344 ± 36 mg/L, p < 0.01), whereas no significant differences were observed for IGA1 (1,788 ± 127 vs 1,974 ± 216 mg/L, p = NS) or IGA (2,277 ± 142 vs 2,319 ± 243 mg/L, p = NS) (Fig. 3A). To further validate the association between IGA2 plasma levels and the occurrence of AAA, we analyzed IGA2 plasma levels in a larger cohort of AAA patients (n = 189) and controls (n = 64) (Table 1). Again, IGA2 plasma levels were significantly increased when compared to controls (551 ± 21 vs 411 ± 26 mg/L, p < 0.001, Fig. 3B). The association between elevated IGA2 levels (medium + high tertiles) and AAA presence was found to be independent of risk factors and treatments [OR = 2.140 (1.109–4.130), p < 0.05, Table 2]. Furthermore, IGA2 was correlated with AAA diameter, a surrogate marker of AAA growth, independently of potential confounding factors (r = 0.13, p < 0.05, Supp. Table 1). Finally, we assess the potential association of IGA2 with AAA progression, showing that elevated IGA2 levels in AAA patients were found to be independently associated with an increased risk of AAA surgery [HR = 1.941 (1.108–3.399), p < 0.05, Table 2, Fig. 3C].

**Table 1:** Clinical characteristics of the validation cohort

	Controls (n = 64)	AAA patients (n = 189)	P value
Age	69.8 ± 2.7	69.9 ± 2.7	ns
BMI (%)	25.7	27.3	** < 0.05**
Hypertension (%)	44	50	ns
Smoking (%)	12	42	** < 0.01**
Diabetes (%)	16	12	ns
Previous CVD (%)	8	21	** < 0.05**
Statins (%)	39	50	ns
Aspirin (%)	27	44	** < 0.05**

**Table 2:** Multivariate analysis of the validation cohort

	OR (95% CI)	P value	HR (95% CI)	P value
Elevated IGA2	2.140 (1.109–4.130)	** < 0.05**	1.941 (1.108–3.399)	** < 0.05**
BMI	1.000 (1.000–1.000)	** < 0.001**	1.000 (1.000–1.000)	** < 0.001**
Smoke	6.618 (2.812–15.576)	** < 0.001**	0.914 (0.568–1.471)	ns
Previous CVD	2.528 (0.842–7.586)	ns	0.983 (0.531–1.820)	ns
Low dose aspirin	1.852 (0.884–3.876)	ns	0.826 (0.499–1.367)	ns

### Polymeric immunoglobulin receptor levels are increased in human AAA wall

**Fig. 4:**
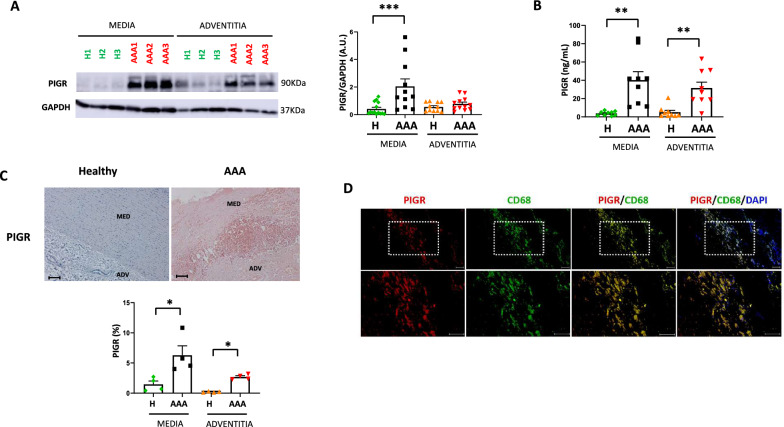
Polymeric immunoglobulin receptor levels are increased in human AAA wall. **A** Western blot and densitometric analysis of PIGR after correction for GAPDH (loading control) in medial and adventitial layers of human AAA and healthy aortas (healthy media n = 14; AAA media n = 11; healthy adventitia n = 11; AAA adventitia n = 12). **B** Quantification by ELISA of PIGR concentration (ng/mL) in the tissue-conditioned media of AAA and healthy aortas (healthy media n = 9; AAA media n = 9; healthy adventitia n = 9; AAA adventitia n = 9). **C** Representative immunohistochemistry and quantification of PIGR in serial aortic cross-sections from AAA and healthy aortas (n = 4 for both). Scale bars, 100 µm. **D** Representative double immunofluorescence staining of PIGR (red) and CD68 (green) in AAA wall. DAPI nuclear staining (blue) is shown. Scale bars, 50 μm. Data represent means ± SEM. Mann–Whitney U test for healthy media vs. AAA media and healthy adventitia vs. AAA adventitia in (**A**) and (**B**). **p < 0.01, ***p < 0.001. A.U., arbitrary units

To ascertain the presence of IGA receptors in the AAA wall of the aorta, we made used of our proteomics data. We observed an increase of polymeric immunoglobulin receptor (PIGR) expression in the medial layer (Supp. Table 2), while PIGR was not detected in the adventitia (Supp. Table 3). Western blot analysis of AAA tissue-homogenates confirmed the significant increase in PIGR levels in the medial layer, while PIGR levels were not significantly changed in the AAA adventitial layer (Fig. 4A). *PIGR* mRNA expression was significantly elevated in both the AAA medial and adventitial layers (Supp. Figure 3). Moreover, PIGR levels were increased in AAA tissue-conditioned media, both in medial and adventitial layers as analyzed by ELISA (Fig. 4B). In addition, immunohistochemistry of PIGR in human AAA tissues revealed a higher expression in the AAA wall compared to healthy aortas (Fig. 4C). Given that PIGR expression has been predominantly observed in epithelial cells, we sought to identify the potential cell types involved in PIGR expression in the human aortic wall. As human AAA medial layer is characterized by an increase in macrophage infiltration and proinflammatory cytokine expression (as observed by increased *CD68* and *TNF-α* mRNA expression, Supp. Figure 3), we tested whether macrophages could contribute to PIGR expression in human AAA. Colocalization studies revealed that PIGR is expressed in CD68^+^ cells within the human AAA wall (Fig. 4D).

### PIGR is expressed and released by human macrophages

**Fig. 5:**
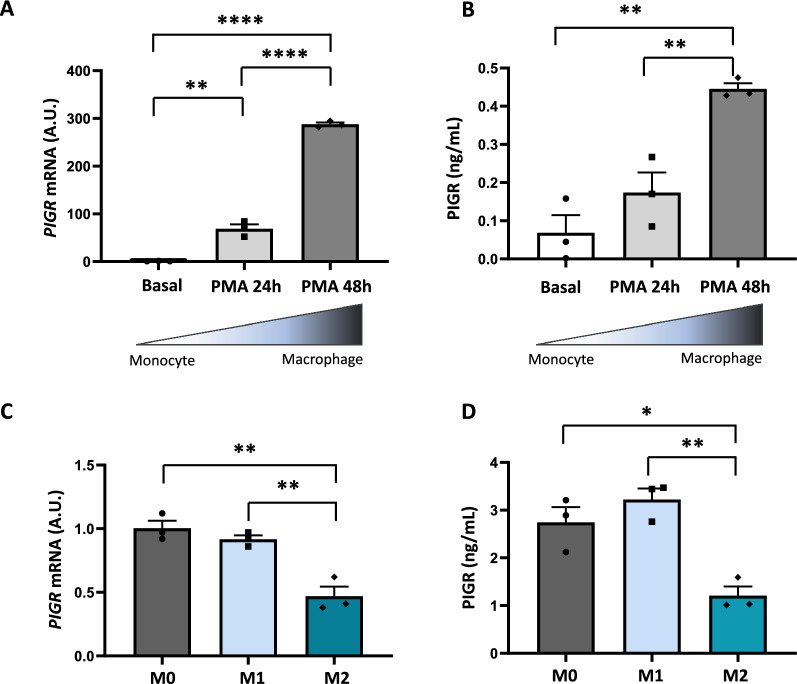
PIGR is expressed and released by human macrophages. **A** Relative *PIGR* mRNA expression of THP-1 monocytes vs. PMA-stimulated THP-1 macrophages (100 nM) at 24 and 48 h upon stimulation. PCR values normalized by *GAPDH* mRNA endogenous control are expressed as fold increases over basal condition at 24 or 48 h. **B** Quantification by ELISA of PIGR concentration (ng/mL) in the tissue-conditioned media of THP-1 monocytes and THP-1 macrophages at 24 and 48 h upon PMA stimulation. **C** Relative *PIGR* mRNA expression of non-polarized THP-1 macrophages (PMA 100 nM for 72 h, M0), polarized M1 macrophages (20 ng/mL IFN-γ + 100 ng/mL LPS, 24 h) and polarized M2 macrophages (20 ng/mL IL-4 + 20 ng/mL IL-13, 24 h). PCR values normalized by *GAPDH* mRNA endogenous control are expressed as fold increases over basal condition at 24 or 48 h. **D** Quantification by ELISA of PIGR concentration (ng/mL) in the tissue-conditioned media of non-polarized THP-1 macrophages (PMA 100 nM for 72 h, M0), polarized M1 macrophages (20 ng/mL IFN-γ + 100 ng/mL LPS, 48 h) and polarized M2 macrophages (20 ng/mL IL-4 + 20 ng/mL IL-13, 48 h). Data represent means ± SEM. Student's t-test. *p < 0.05, **p < 0.01, ****p < 0.0001. A.U., arbitrary units

To further confirm the expression of PIGR by macrophages, we tested *PIGR* mRNA expression and PIGR release by human THP-1-derived macrophages in vitro. THP-1 monocyte differentiation into macrophages with PMA for 24 to 48 h resulted in an increase in *PIGR* mRNA expression (Fig. 5A), as well as PIGR release at 48 h (Fig. 5B). We stimulated THP-1-derived macrophages with different doses of IGA (IHUIGA5MG Innovative Research, 1,10 and 100 ug/mL) during 24 h. However, we did not observe any effect on PIGR mRNA expression at any of the doses tested (not shown). In contrast, TNF-α induced *PIGR* mRNA expression in THP-1-derived macrophages (Supp. Figure 3). To test whether *PIGR* expression could be associated with different polarization states, mainly M1 and M2, THP-1 monocytes were differentiated into macrophages with PMA for 72 h and then incubated with IFN-γ and LPS for M1 polarization or with IL-4 and IL-13 for M2 polarization. We found that *PIGR* mRNA expression and release were decreased in M2 macrophages compared to M1 macrophages at 24 and 48 h, respectively (Fig. 5C and D), indicating that *PIGR* expression is regulated during macrophage polarization.

### *Pigr* deficiency in the hematopoietic compartment decreases AAA incidence, size and macrophage infiltration.

**Fig. 6:**
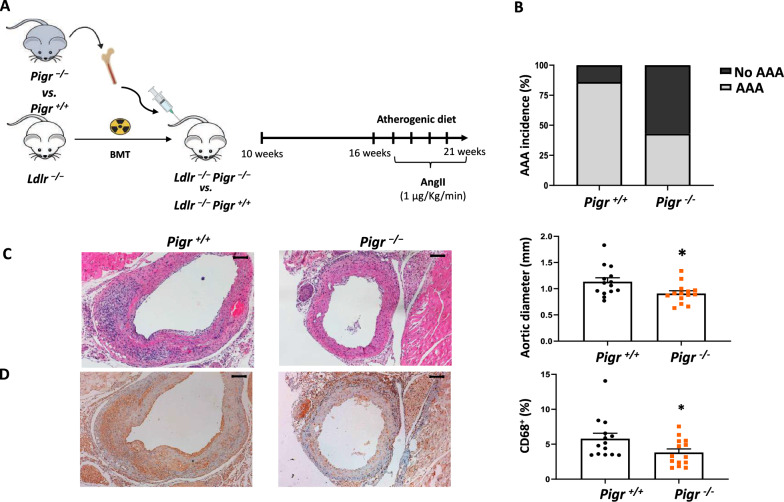
Pigr deficiency in the hematopoietic compartment decreases AAA development. **A** Experimental design and (**B**) incidence of AAA in *Ldlr*^*−/−*^* Pigr*^+/+^ (n = 14) and *Ldlr*^*−/−*^* Pigr*^*−/−*^ (n = 14) chimeras at 28 days after subcutaneous AngII infusion via osmotic pumps. **C** Representative H&E staining and quantification of the maximal abdominal aortic diameter. **D** Representative photographs and quantification of CD68 immunostaining. Data represent means ± SEM. *p < 0.05, Mann–Whitney U test. Scale bars, 100 µm

To assess the hematopoietic involvement of PIGR in AAA, a BM transplantation model was then carried out in the AngII model of AAA. In this respect, *Ldlr*^*−/−*^ mice (n = 28) mice were irradiated and transplanted with BM cells from *Pigr*^*−/−*^ (n = 14) or *Pigr*^+/+^ mice (n = 14) (Fig. 6A). After 4 weeks, flow cytometry analysis of CD45 allelic variants confirmed the successful BM reconstitution (Supp. Figure 4). Mice then were fed a Western-type diet and infused with AngII for 28 days. No differences were found in body weight nor in serum lipid concentrations between genotypes (Supp. Figure 4). Interestingly, only 2/14 (14%) *Ldlr*^*−/−*^* Pigr*^*−/−*^ chimeras developed AAA compared to 8/14 (57%) *Ldlr*^*−/−*^* Pigr*^+/+^ chimeras (p < 0.05, Fig. 6B). The maximal abdominal aortic diameter on day 28 in *Ldlr*^*−/−*^* Pigr*^*−/−*^ chimeras was significantly lower than that in *Ldlr*^*−/−*^* Pigr*^+/+^ chimeras (0.91 ± 0.05 vs 1.13 ± 0.07 mm, p < 0.05, Fig. 6C). Regarding macrophage infiltration, there was a prominent decrease in CD68^+^ cells in *Ldlr*^*−/−*^* Pigr*^*−/−*^ chimeras (3.5 ± 0.5 vs 5.6 ± 0.7%, p < 0.05, Fig. 6D). In contrast, we did not observe significant differences α-SMA staining between *Pigr*^+/+^ and *Pigr*^*−/−*^ chimeras (8.2 ± 1.1 vs 10.8 ± 1.1, p = ns). Overall, these data suggest that *Pigr* deficiency in the hematopoietic lineage decreases AAA formation and incidence associated with a lesser wall macrophage infiltration.

## Discussion

In this study, we conducted a compartment-resolved proteomic analysis of human AAA and control aortic wall with the aim of identifying novel mediators involved in AAA. A comparison of the medial and adventitial layers of AAA walls revealed a greater number of significantly altered proteins in the adventitial layer, which may be associated with the higher cell heterogeneity observed in the pathological adventitia. The differentially regulated processes between the AAA medial and adventitial layers included cellular detoxification, platelet degranulation and actin organization in the medial layer, and complement activation, negative regulation of proteolysis and lipid transport in the adventitial layer. The increased antioxidant response observed in the AAA media compared to adventitia is consistent with the higher oxidative stress observed in the adventitial layer [20]. Despite using a compartmentalized proteomic approach to define the distinct contributions of the AAA media and adventitial layers, several pathways were found to be shared between the two layers. Proteins that were upregulated in both compartments were predominantly associated with the immune response. Conversely, common downregulated processes in both, medial and adventitial layer, were primarily related to cell adhesion. The increased abundance of genes related to adaptive and innate immunity in both layers is consistent with the findings from a previous transcriptomic analysis of human AAA walls [21]. Another microarray study conducted on perivascular adipose tissue (PVAT), which surrounds and regulates the adventitial layer, revealed that genes associated with the adaptive immune response were also significantly enriched in AAA [22]. Regarding the downregulated proteins, those related to the contractile phenotype of VSMC such as calponin or α-SMA were observed in AAA walls, in agreement with the importance of VSMC phenotypic switch in AAA [23]. In conclusion, both differential and shared pathways were observed in medial and adventitial layers, suggesting the potential contribution of proteins from extracellular sources. In this regard, our previous research has demonstrated that a significant portion of the aortic proteome may originate from the systemic retention of proteins within atherosclerotic plaques [24]. Given the significant contribution of extracellular sources in the aortic proteome and the cell heterogeneity of adventitial AAA, a single-cell proteomic analysis of human AAA is likely to improve our understanding on the specific cell type contributions to the AAA phenotype. To date, only a limited number of studies have employed single-cell RNA sequencing, but not proteomic, analysis of human AAA [25–27].

The proteomic analysis of human AAA wall revealed that IGA and IGG subclasses were the most significantly upregulated proteins. A previous transcriptomic study in human AAA wall [19] did not identify IGA or IGG subclasses, which may be attributed to the different technical approach employed or the potential systemic source of the proteins. However, one of the most significantly upregulated genes in that study was J-chain, a pivotal protein involved in polymerization of IGA (and IgM) and expressed by B/plasma cells [28]. In this respect, IGA^+^ plasma cells have been found in non-mucosal tissues such as the adventitia of the vascular wall [29]. Interestingly, *IGHA1*, along with J-chain, was identified in B cells by single-cell RNA seq analysis of human control and AAA tissues [25]. In agreement, our reanalysis of a previous single-cell RNA seq study [27] revealed the potential involvement of aortic B/plasma cells in *IGA1/2* mRNA expression (not shown). However, due to the huge amount of IGA protein detected in our study, we can anticipate that a fraction of the IGA1 and IGA2 proteins are originally produced in distant locations (most likely the gut) and then retained in AAA tissue, as previously observed for other high-abundant plasma proteins [6, 7]. While the role of other antibodies, such as IGG and IGE, has been previously addressed in AAA [30], the contribution of IGA (and/or IGA isotypes) remains unknown. Differences between mice and humans could have contributed to the scarce knowledge of IGA in vascular pathologies. In contrast with humans, who express the two IGA1 and IGA2 isoforms, mice only have one IgA subclass. Besides, serum IgA in mice is mainly polymeric, while in humans is monomeric, and there are no mouse homologues of the human Fc alpha receptor (FcαRI) [31]. IGA is the most abundant immunoglobulin in the human body, with a primary expression in mucosal surfaces, where it is released as secretory IGA (sIGA). However, although the majority of IGA is mainly generated in gut-associated lymphoid tissues, IGA-producing plasma cells have been observed in various pathological tissues, including the vasculature [32]. The traditional view of IGA as a protective antibody due to its role in preventing infections of mucosal tissues (based on the opsonizing effects of sIGA) is being challenged by recent data demonstrating the impact of IGA on immune-inflammatory responses [33–35]. Moreover, IGA functions also depend on the IGA subclasses and isoforms. A recent study demonstrated that IGA2, but not IGA1, induces IL-8 and TNF-α release by macrophages [36]*.* Another publication demonstrated that dimeric IGA2 isoforms elicited a higher dendritic cell activation when compared to monomeric IGA1/2 or dimeric IGA1 [37]. The data in our work suggests that IGA accumulation in the aortic wall may contribute to AAA development by different pathological mechanisms. Moreover, modulation of IGA actions/functions at the aortic level represents a potential therapeutic alternative for the prevention and/or stabilization AAA. Although this hypothesis has yet to be tested in AAA models, genetic germline deletion of IGA significantly reduced inflammation and aortic dilatation in a model of vasculitis induced by *Lactobacillus casei* in mice [38].

The search for novel circulating biomarkers that could help in the diagnosis and more importantly, the prognosis of AAA, represents a field of active research [39–41]. Previous studies have analyzed biomarkers associated with known AAA pathological mechanisms, including proteolysis, oxidative stress or thrombosis [42–44]. Similarly, biomarkers associated with the immune and/or humoral response have been evaluated. Increased levels of IGE in tissue and plasma from AAA patients have been recently described [45]. In this regard, antibodies of the IGA isotype are increasingly being associated with various disorders, including rheumatoid arthritis and IGA nephropathy, among others [46]. Similarly, previous studies have tested IgA antibodies against *Chlamydia pneumoniae* [47] or malondialdehyde-acetaldehyde [48] in AAA plasmas, demonstrating their potential as circulating biomarkers of AAA. Given the observed increase in IGA accumulation in AAA tissues, we thus sought to determine whether this was accompanied by an increase in systemic IGA levels in AAA patients. Our data demonstrate an elevation in plasma levels of IGA2 in AAA patients compared to controls, whereas no significant differences were observed in IGA1 or total IGA levels. Similarly, an increase in plasma levels of IGA2, but not IGA1, has been observed in individuals with subclinical atherosclerosis [49]. Given that IGA1 is the predominant subclass in human blood, fluctuations in IGA1 levels exert a significant influence on total IGA plasma levels. This underscores the importance of examining IGA subclasses to assess their potential as biomarkers of immune-inflammatory diseases. Furthermore, the association of IGA2 with AAA was validated in a larger cohort, where it was confirmed that the association was independent of traditional risk factors and drug therapies. Our findings also indicate that individuals with elevated IGA2 levels exhibit an almost two-fold increased risk of achieving the requisite aortic diameter for surgical intervention. Thus, IGA2 could be potentially used to stratify those patients with a closer follow-up to prevent a sudden rupture during surveillance. For potential implementation in the management of AAA patients, it is important to note that IGA2 levels have been measured in the present study using standard commercial kits routinely used in clinical practice in a fast and economic way; however, its potential use in clinics is hampered by their limited specificity and sensitivity as depicted in the ROC curve of IGA2 for AAA surgery (not shown). Thus, at present, the potential relevance of IGA2 is more as a disease/pathogenic biomarker rather a clinical tool to assess the progression of AAA patients.

IGA exerts its actions via several receptors, including its primary receptor FcαRI [50]. However, IGA can also interact with transferrin receptor 1 (CD71), asialoglycoprotein receptor (ASGPR), Fcα/μR, PIGR and DC-SIGN/SIGNR1 [33]. Among these, here we describe the presence of PIGR in human AAA wall for the first time. The primary function of PIGR is the transport of dimeric IGA and polymeric IGM from the lamina propria across the epithelial barrier to mucosal surfaces [51]. Although PIGR expression was originally thought to be restricted to epithelial cells of the gut, PIGR has also been observed in lung, liver and renal epithelial cells, as well as in cancer cells of different tissue origins [52]. Furthermore, PIGR expression was identified in brain endothelial cells as a potential receptor for *Pneumococc*us entry into the brain [53, 54]. A recent study described increased PIGR and IGA expression in the aorta in a vasculitis model in mice [38]. It is noteworthy that we observed not only PIGR protein expression and secretion in human AAA wall, but also *PIGR* mRNA expression. This suggests the potential synthesis of PIGR by cells present within the pathological tissue. The immunohistochemical analysis of human AAA tissue corroborated the increased PIGR expression, which colocalized with CD68^+^ cells. To further assess the potential contribution of macrophages to PIGR synthesis and secretion by the human AAA wall, human THP-1 monocytes were differentiated into macrophages with PMA. This confirmed that THP-1-derived macrophages were able to express and secrete PIGR into the extracellular medium. Furthermore, we observed that this pattern of expression and secretion of PIGR was associated with a classical M1 pro-inflammatory macrophage phenotype, which has been linked to AAA [55]. In this regard, several immunological factors including IL-1, IL-17, IFN-γ and TNF-α upregulate the expression of PIGR [56]. Accordingly, we observed that TNF-α induced *PIGR* expression in THP-1-derived macrophages. A previous study described that IGA1/2 either alone or in the form of immune complexes induced FcαRI expression in dendritic cells [57]. However, we did not show any effect of IGA (containing both IGA1 and 2) on *PIGR* mRNA expression at any of the doses tested in human macrophages, which could suggest that the ligand is not involved in its receptor regulation. To further assess the potential effect of PIGR in AAA, we analyzed the hematopoietic contribution of PIGR in the AngII model of AAA in mice. Interestingly, *Pigr* deletion in the hematopoietic compartment reduced AAA size, incidence and macrophage infiltration, suggesting a pathogenic role for PIGR in AAA development, potentially linked to inflammatory mechanisms. In this respect, silencing *Pigr* alleviated symptoms, reduced IL-33 expression and restrained hepatic Th2 inflammation in a biliary atresia mouse model [58]. Nevertheless, further investigation is required to ascertain whether PIGR plays a role in the pathological mechanisms associated with AAA.

Overall, our study provides an unbiased insight into the pattern of proteins altered in the human AAA wall, identifying novel potential pathogenic biomarkers and/or mediators of AAA, including IGA and PIGR. Notably, *Pigr* deletion in the hematopoietic compartment decreased AAA incidence, suggesting potential therapeutic applications to prevent AAA.

## Limitations of the study

Regarding the examination of IGA2 in the plasma of patients with AAA, the principal strength of this study is the population-based design of the VIVA trial, which has an exceptionally high attendance rate, thereby reducing the risk of selection bias. However, not all diagnosed cases had samples available, leaving a risk of selection bias. In addition, a systematic approach was used to identify confounders, but in the end, residual confounding by nature is always a risk in observational studies. In addition, cumulative average estimates of IGA2 would provide a more robust association with AAA progression than a single baseline measurement. Finally, since the present study was conducted using a single plasma cohort, further validation in additional cohorts, including samples from different sexes, is needed to confirm the results.

## Supplementary Information

Additional file 1. Table 1.- linear regression analysis of iga2 with aortic diameter

Additional file 2. Table 2.- list of proteins identified by lc-ms in aaa medial layer

Additional file 3. Table 3.- list of proteins identified by lc-ms in aaa adventitial layer

Additional file 4. Figure 1.- diagram of the study setup design. Figure 2.- functional enrichment analysis of proteins significantly increased in aaa medial and adventitial layers. Figure 3.- mrna expression analysis in homogenates of medial and adventitial layers of control aortas and aaa and thp-1-derived macrophages. Figure 4.- bm reconstitution, body weight and lipid profile of the animal model

## Data Availability

The main data generated or analyzed during this study are included in this manuscript and its additional files. The mass spectrometry proteomics data have been deposited to the ProteomeXchange Consortium via the PRIDE [59] partner repository with the dataset identifier PXD056789.

## Abbreviations

AAA: Abdominal aortic aneurysm
PIGR: Polymeric immunoglobulin receptor
IGHA1: Immunoglobulin heavy constant A1
IGHA2: Immunoglobulin heavy constant A2
IGHG1: Immunoglobulin heavy constant G1
IGHG2: Immunoglobulin heavy constant G2
IGHG3: Immunoglobulin heavy constant G3
IGHG4: Immunoglobulin heavy constant G4
IGA1: Immunoglobulin A1 (including both, heavy and light chains)
IGA2: Immunoglobulin A2 (including both, heavy and light chains)
VSMC: Vascular smooth muscle cells
CVD: Cardiovascular disease
SEM: Standard error of the mean
FDR: False Discovery Rate
PVAT: Perivascular adipose tissue
