# Supplementary material for: Immunoglobulin A/PIGR axis as potential mediators of human abdominal aortic aneurysms revealed by topologically resolved proteomics
Source: J Transl Med. 2025 Jul 7;23:747. doi: 10.1186/s12967-025-06758-y (PMC12232777; doi:10.1186/s12967-025-06758-y)
Supplement: Supplementary file 4 — Additional file 4. Figure 1.- diagram of the study setup design. Figure 2.- functional enrichment analysis of proteins significantly increased in aaa medial and adventitial layers. Figure 3.- mrna expression analysis in homogenates of medial and adventitial layers of control aortas and aaa and thp-1-derived macrophages. Figure 4.- bm reconstitution, body weight and lipid profile of the animal model [file 12967_2025_6758_MOESM4_ESM.pdf]

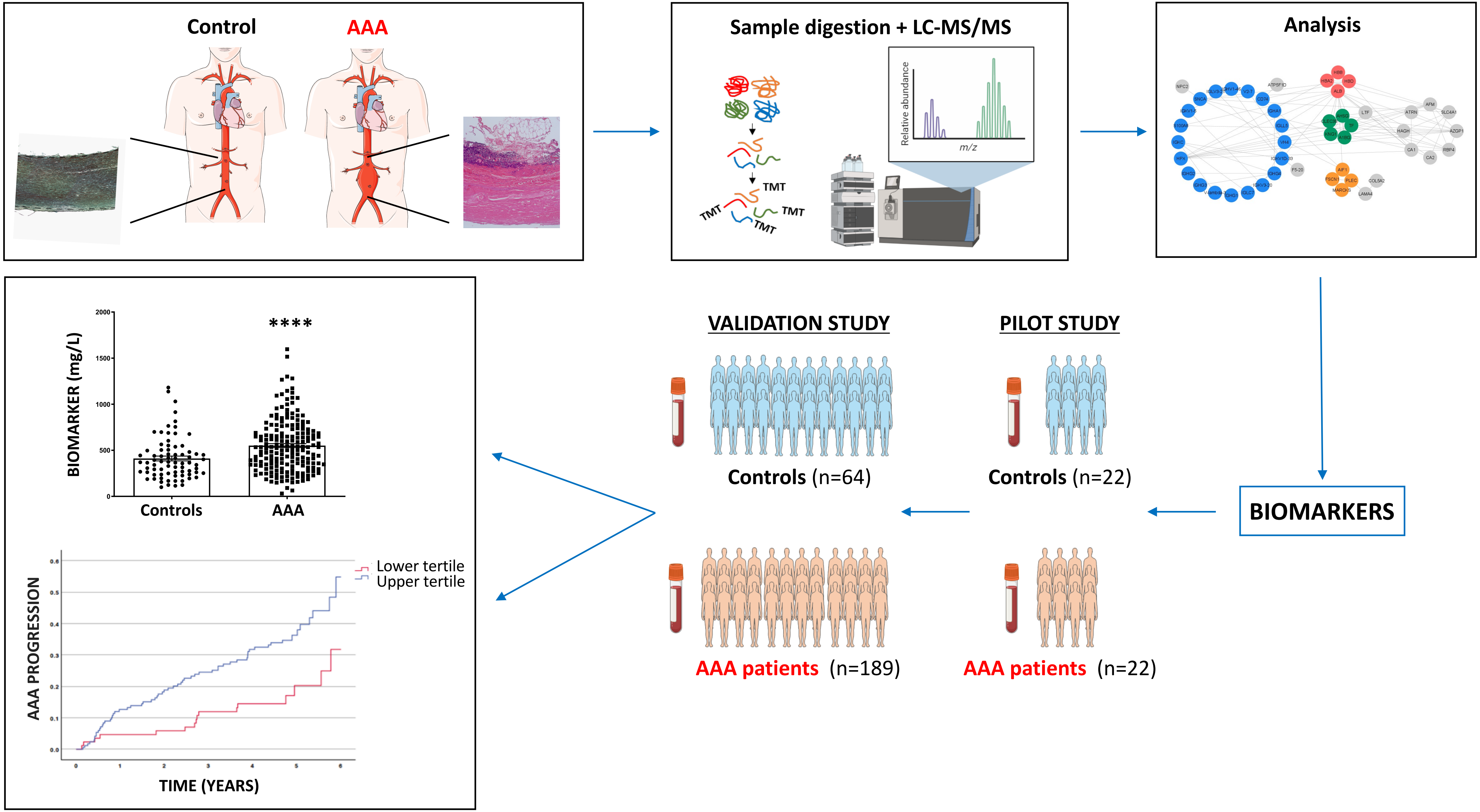

Supp. Figure 1. Experimental design.

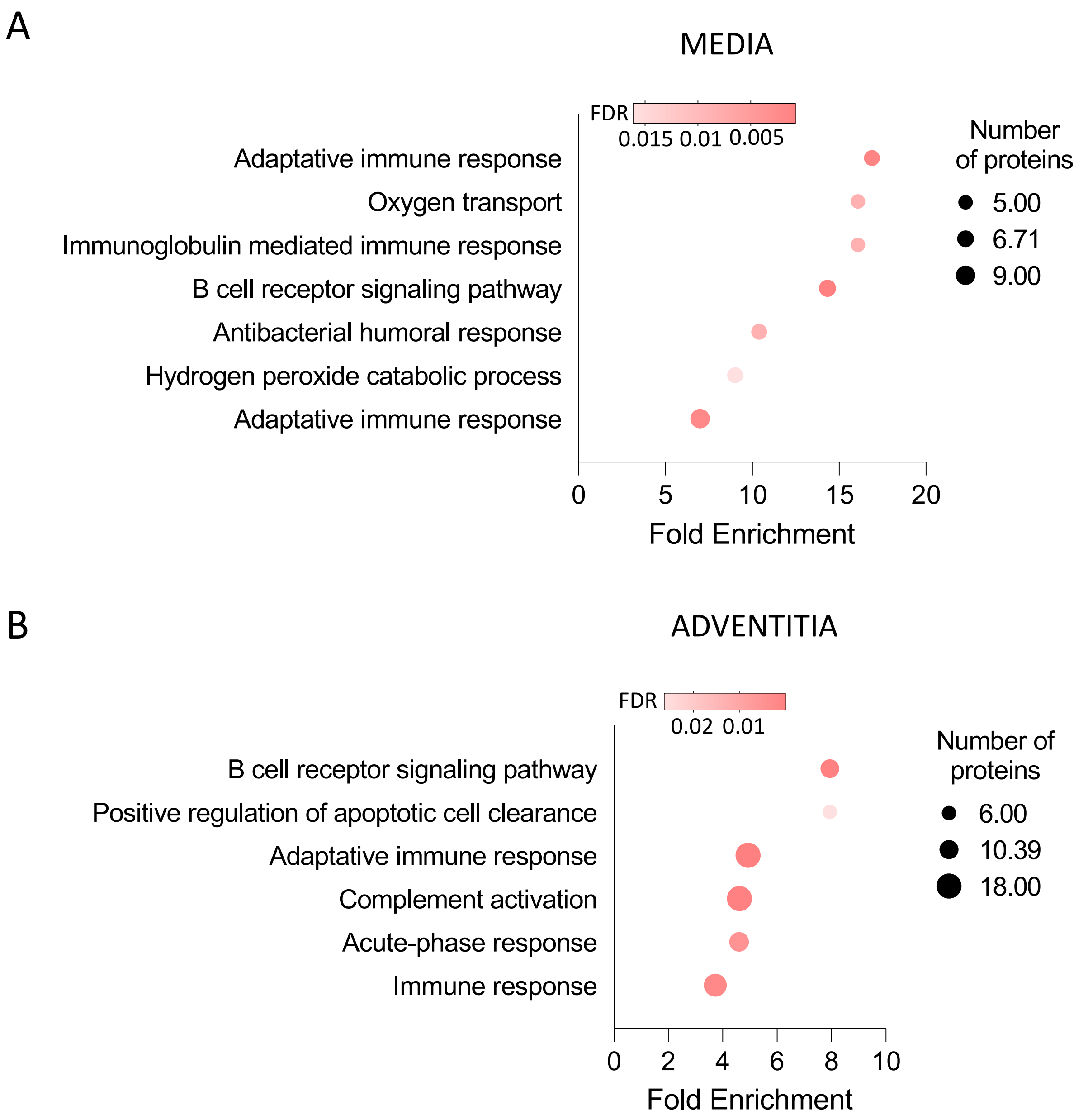

Supp. Figure 2. Functional enrichment analysis of proteins significantly increased (FDR<0.05) in AAA medial (A) and adventitial (B) layers. Points represent category fold enrichment and point size indicates the number of proteins belonging to each category. The colour of the points represents the FDR values of category enrichment.

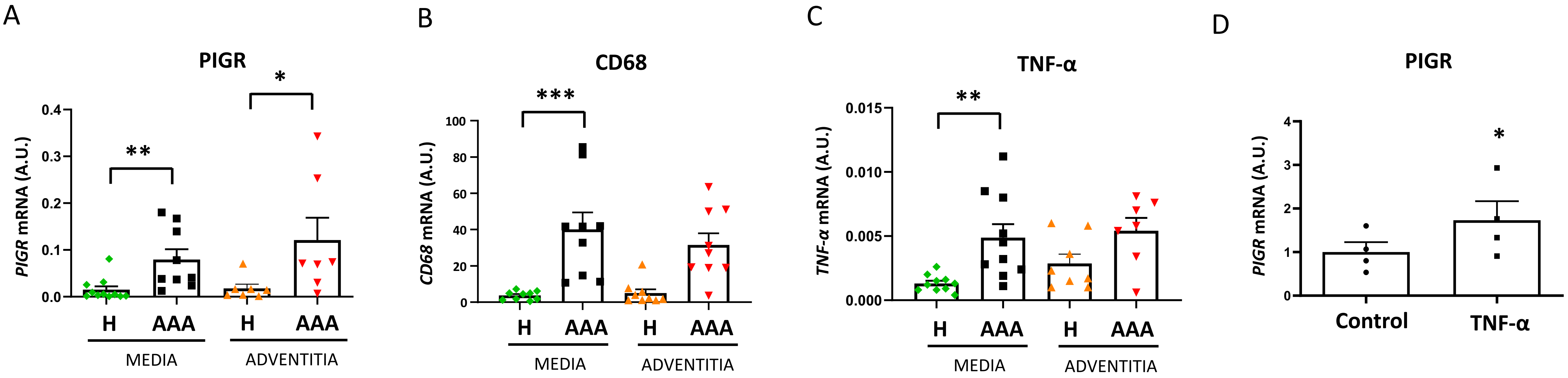

Supp. Figure 3. mRNA analysis of healthy and AAA aortas (A-C) and of THP-1-derived macrophages stimulated with TNF-α (D).

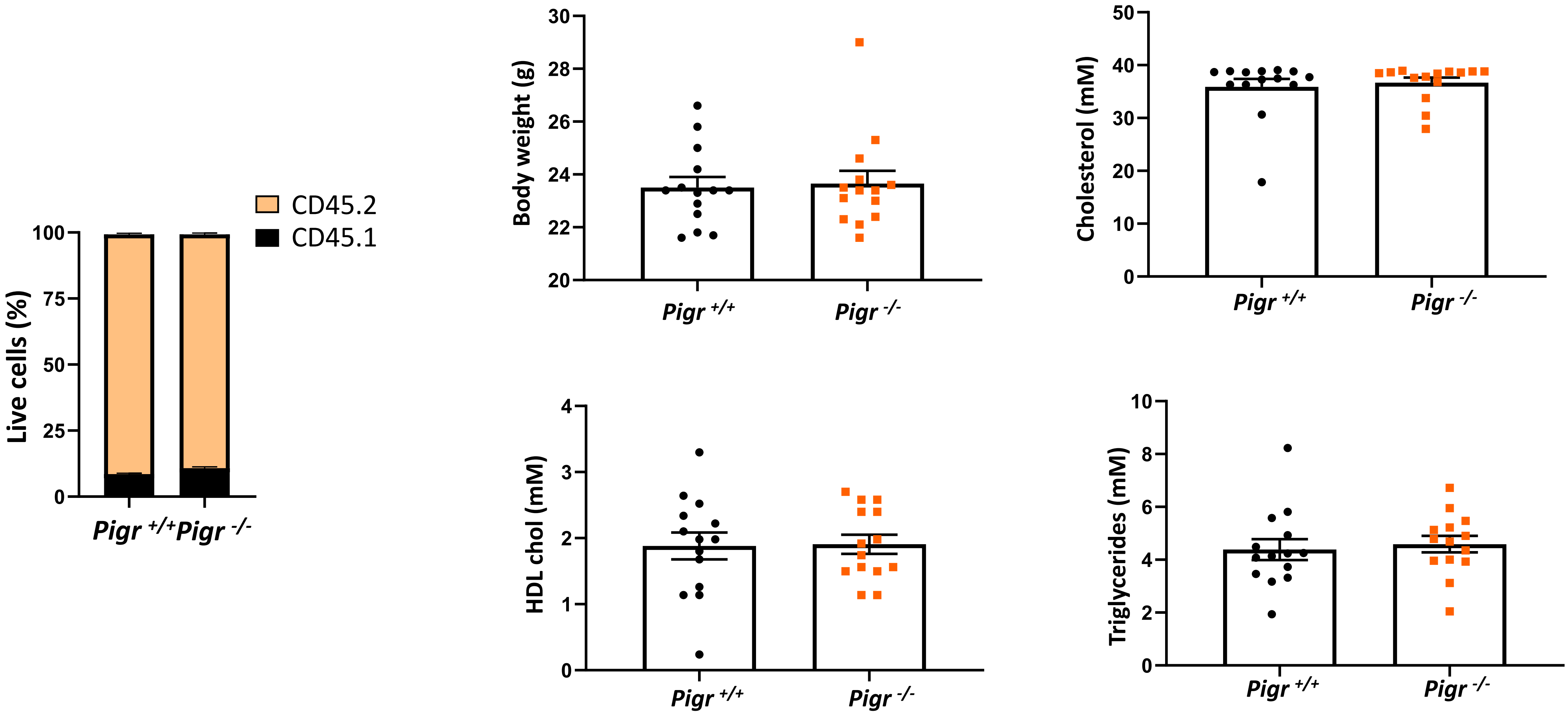

Supp. Figure 4. BM reconstitution, body weight and lipid profile of the animal model.
